# The three-hybrid genetic composition of an Ecuadorian population using AIMs-InDels compared with autosomes, mitochondrial DNA and Y chromosome data

**DOI:** 10.1038/s41598-019-45723-w

**Published:** 2019-06-25

**Authors:** Ana Karina Zambrano, Aníbal Gaviria, Santiago Cobos-Navarrete, Carmen Gruezo, Cristina Rodríguez-Pollit, Isaac Armendáriz-Castillo, Jennyfer M. García-Cárdenas, Santiago Guerrero, Andrés López-Cortés, Paola E. Leone, Andy Pérez-Villa, Patricia Guevara-Ramírez, Verónica Yumiceba, Gisella Fiallos, Margarita Vela, César Paz-y-Miño

**Affiliations:** 1 Centro de Investigación Genética y Genómica, Facultad de Ciencias de la Salud Eugenio Espejo, Universidad UTE, Av. Mariscal Sucre and Mariana de Jesús, Block I, Quito, 170129 Ecuador; 2 Laboratorio de Genética, Centros Médicos Especializados Cruz Roja Ecuatoriana, Papallacta oe1-66, Quito, 170512 Ecuador

**Keywords:** Structural variation, Haplotypes

## Abstract

The history of Ecuador was marked by the arrival of Europeans with Africans, resulting in the mixture of Native Americans with Africans and Europeans. The present study contributes to the knowledge of the Ecuadorian mestizo population by offering information about ancestry and ethnic heterogeneity. Forty-six AIM-InDels (Ancestry Informative Insertion/Deletion Markers) were used to obtain information on 240 Ecuadorian individuals from three regions (Amazonia, the Highlands, and the Coast). As a result, the population involved a significant contribution from Native Americans (values up to 51%), followed by Europeans (values up to 33%) and Africans (values up to 13%). Furthermore, we compared the data obtained with nine previously reported scientific articles on autosomal, mitochondrial DNA and Y chromosomes. The admixture results correspond to Ecuador’s historical background and vary slightly between regions.

## Introduction

The origin of men in America begins with their arrival from Asia by the Bering Strait, which started forty or fifty thousand years before Christ^1^. Specifically, in “Ecuatorial Andinoamérica” (known today as Ecuador), there is evidence of settlement from twelve thousand years ago^1^. American history continues with the arrival of Europeans (Spanish) to the continent, in which African descendants were brought as slaves^2^. Consequently, the Spaniards took control of the Panama Isthmus and then started to travel south. The first conquer and colonization expedition to Ecuador was in 1526. They arrived at the coast where they disembarked to go inland^1^.

In addition, slaves arrived from Africa to Europe due to the slave trade, in addition to Muslim and Portuguese as merchants, since the Middle Ages. As a result, the slaves who came to America constituted a heterogeneous group from different societies and cultures^3,4^.

For approximately twelve thousand years, the lands that are now known as Ecuador have been populated by indigenous people from Asia and Oceania. Nonetheless, after the conquering, the Spanish brought their language and customs, and the population started mixing between the natives and the Spanish and the African slaves, resulting in diversity in the Ecuadorian population^5^.

The census in Ecuador that began in 1950 classified people by asking for certain characteristics, such as language, and tried to predict the behavior of the indigenous groups^6,7^. The most recent census was in 2010, and according to the results, the population projection for 2019 was an estimated 17 267 986 Ecuadorians, with 24 provinces distributed in 4 different regions: the Coast (8 523 453), the Highlands or the Andes (7 733 725), Amazonia (937 406), and the insular region (32 320)^8^. Moreover, in the census, Ecuadorians self-identified as “mestizos” 71.9%, “montubios” 7.4%, Afro-Ecuadorians 7.2%, “Indígenas” 7% and “blancos” 6.1%^9^.

The genetic population structure of Ecuador has been previously studied using different genetic markers, such as mitochondrial DNA^10–12^, Y chromosome^10,13^ or autosomes^14–18^. Some of these studies are focused on Native American or Afro-Ecuadorian groups^10–12^, and others are focused on mestizo populations but with a sampling that did not consider the Ecuadorian regions^14–18^. Moreover, those reports emphasize different genetic markers, such as AIMs (Ancestry Informative Markers), that have been used to fully understand the differentiation (ancestral or geographical) across populations, which can also infer migration, admixture, colonization, and/or invasion events^19^. Here, we report genetic data of the Ecuadorian mestizo population using AIMs-InDels. In addition, we analyzed and compared the data with previously reported data of the genetic ancestry of the Ecuadorian population.

## Materials and Methods

### Sample collection and DNA extraction

A total of 240 unrelated self-identified mestizo samples were randomly selected (53 from all of the provinces in Amazonia, 88 from all of the provinces in the Highlands, and 99 from all of the provinces on the Coast). All individuals signed the informed consent form for population genetic studies. Blood samples were collected on FTA paper (GE Healthcare Life Sciences) at the Genetic Laboratory of Centros Médicos Especializados Cruz Roja Ecuatoriana.

DNA was extracted using Chelex 100 (Bio-Rad) (20%) according to the standardized method of the Genetic Laboratory based on the method published by the President´s DNA Initiative^20^ protocol, with a modification in the concentration of Chelex used (20%).

All methods were carried out in accordance with relevant guidelines and regulation^21^. Moreover, the experimental protocols were approved with the number 2018-127E by “Comité de Ética de Investigación en Seres Humanos Universidad San Francisco de Quito”.

### Amplification and genotyping

PCR amplification of the 46 AIM-InDels was performed with the same primers and one multiplex reaction, according to Pereira *et al*.^22^. Fragment separation and detection were executed on the ABI PRISM 3100, 3130 and 3500 Genetic Analyzers (Applied Biosystems). The results were collected with Data Collection v2.0 and v4.0 and analyzed by Gene Mapper v3.2 and v5 (Applied Biosystems). Along with the samples, to evaluate the amplification efficiency during the PCR and the genotyping steps, positive controls were used (male DNA control 007^23^, female DNA control 9947A^24^ and random references samples from GHEP^25^ quality control to evaluate the amplification process), and one negative control was used to test for contamination. Short alleles were coded as 1, while long alleles were coded as 2.

### Statistical analyses

The population genetic parameters (allele frequencies, Hardy-Weinberg equilibrium, and F_ST_ genetic distances) were estimated using Arlequin v3.5.2.2^26^. With STATISTICA v.13^27^, an multidimensional scaling (MDS) scatterplot was constructed to visualize the genetic distances between Ecuador and each reference population from HGDP-CEPH (Africans, Europeans and Native Americans) subset H952^21,28,29^. Once the allele frequencies were obtained, the allele frequency differentials (δ) were estimated by comparing the frequencies reported by Pereira *et al*.^22^ from Native Americans, Europeans, and Africans due to the historical background of Ecuador.

Ancestry inferences were made using STRUCTURE v2.3.4^30^; the runs consisted of a burn-in length of 5 000 followed by 5 000 Markov Chain Monte Carlo (MCMC) interactions. The option used was the admixture model (“Use population information to test for migrants”). Moreover, three repetitions were performed to estimate the cluster used, and the k value was tested from K = 1 to K = 20 to evaluate the Ln probability of data (LnP(D)) by plotting it according to Evanno *et al*.^31^. Finally, principal component analysis (PCA) was performed to visualize the population structure: the relationship between the Ecuadorian population and the reference population used^32^. RStudio v1.0.44^33^ was used to obtain the PCA plot and the variance in the two first principal components.

### Comparative analysis with previously reported data

An exhaustive study was performed to compare the present study with the results obtained in previous reported studies that used autosomes, Y chromosome or mitochondrial DNA related to the origin of the Ecuadorian population. Table 1 shows the previously reported data that are used in the current work.

**Table 1:** Genetic markers, populations and references used in the review of origin of Ecuadorians.

Genetic markers used	Population under study	Total number of populations	Reference
Mitochondrial DNA	Waoranis	36	^10^
Mitochondrial DNA	Kichwas and Mestizos	107	^11^
Mitochondrial DNA	Cayapas	204	^12^
Y- STRs	Mestizos	415	^13^
Autosomal AIMs	Mestizo, Kichwas, Afro-Ecuadorians	162	^14^
Autosomal AIMs-InDels	Mestizos	171	^15^
Autosomal SNPs	Kichwas and Mestizos	119	^16^
Autosomal SNPs	Mestizos	19	^17^
Autosomal SNPs	Mestizos	6	^18^

## Results

### Population genetic parameters

Allele frequencies for short alleles (1) and long alleles (2) were estimated for the 240 samples from the three regions (Amazonia (AM), Highlands (HL), Coast (CO)) and joined as Ecuador (ECU). Moreover, observed and expected heterozygosities were calculated for Ecuador, in which the marker with the highest variability was MID-397 (0.551). Allele frequency differentials (δ) were estimated for Ecuador against each reference population (Africans, Europeans and Native Americans), resulting in a differential average of 0.320 with Africans, 0.196 with Europeans, and 0.12 with Native Americans. As expected, Ecuador has a similar level of diversity with the reference Native American population. Furthermore, allele frequency differentials (δ) were also estimated between regions, showing a low allele frequency differential mean (0.045 Amazonia-Highland; 0.071 Amazonia-Coast; 0.055 Highland-Coast) that increased when the geographical distance increased. (Table S1)

Once the Bonferroni correction (p > 0.001) was applied, no significant deviations from Hardy-Weinberg equilibrium were found for the 46 loci in the population under study. Moreover, linkage disequilibrium (α > 4.6 × 10-5) did not show any significant associations between the markers, except for MID-225 and MID-94 in the Coast population, but not in the other two populations. That result suggests that there is not a real association between the markers, allowing the use of the migration model in STRUCTURE analysis^34^.

### Genetic distances

The data obtained from the Ecuadorian population were used to estimate the F_ST_ (Table 2) genetic distances between all population pairs.

**Table 2:** Genetic distances (F_ST_) between the Ecuadorian population (Amazonia, the Highlands, and the Coast) and the reference population (Africa, Europe and Native America) (lower diagonal) and P values (upper diagonal).

		Amazonia	Highland	Coast	Africa	Europe	Native America
Ecuador	Amazonia	—	0.0201	<5e-05	<5e-05	<5e-05	<5e-05
	Highland	0.00279	—	<5e-05	<5e-05	<5e-05	<5e-05
	Coast	0.01220	0.00666	—	<5e-05	<5e-05	<5e-05
Reference	Africa	0.35975	0.34842	0.28422	—	<5e-05	<5e-05
	Europe	0.16233	0.15013	0.11830	0.36515	—	<5e-05
	Native America	0.05140	0.05218	0.07399	0.44273	0.29768	—

**Figure 1:**
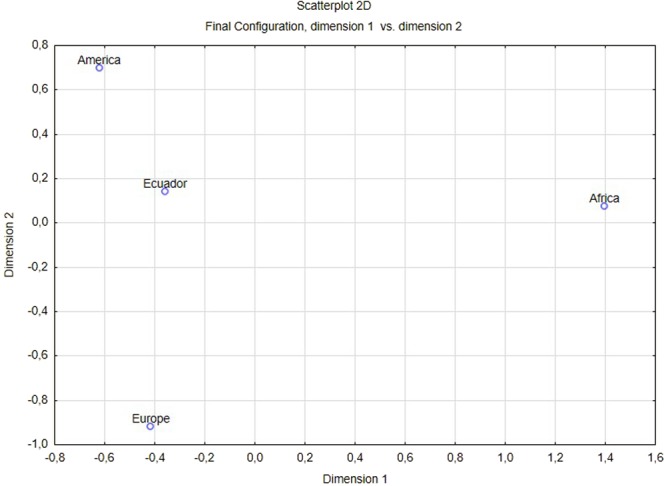
Multidimensional scaling plot from the F_ST_ between the Ecuadorian population and the reference population.

The pairwise genetic distances showed significant differentiation between all Ecuadorian samples and the reference population and between Ecuadorian regions. To interpret the genetic distances obtained, we used the suggested values classification of Ballaux *et al*. (2002). Values between 0–0.05 indicate little genetic distance, 0.05 and 0.15 indicate moderate differentiation and 0.15 and 0.25 indicate great differentiation, and values up to 0.25 indicate very great differentiation^35^. In the present study, there is a small genetic distance between all the Ecuadorian regions (Amazonia-Highland, Amazonia-Coast and Highland-Coast). When compared with the reference population, there is a moderate differentiation between all Ecuadorian regions and the Native American population with values < 0.073, and between the Highland and Europe populations (0.15) and the Coast and Europe populations (0.118), there is a great differentiation between African and all the Ecuadorian regions and between Europe and Amazonia. The pairwise F_ST_ is represented in the multidimensional scaling plot in Fig. 1 to visualize the level of similarities between Ecuadorian and the reference population. In the plot, the Ecuadorian sample appears closer to the Native American reference sample.

Moreover, a population pairwise F_ST_ was performed to obtain the genetic distances between the Ecuadorian population and available published data from the South American population. The evaluations were obtained from Colombia^36^ (F_ST_ = 0.014, p < 5e-05), which is located in the north border of Ecuador and Brazil^19,37^ (F_ST_ = 0.054, p < 5e-05), which is located further away. Those results showed little differentiation with Colombia and were moderately differentiated with Brazil (Fig. S1), validating the geographic distances and the difference in history of each population.

### Admixture analysis

**Figure 2:**
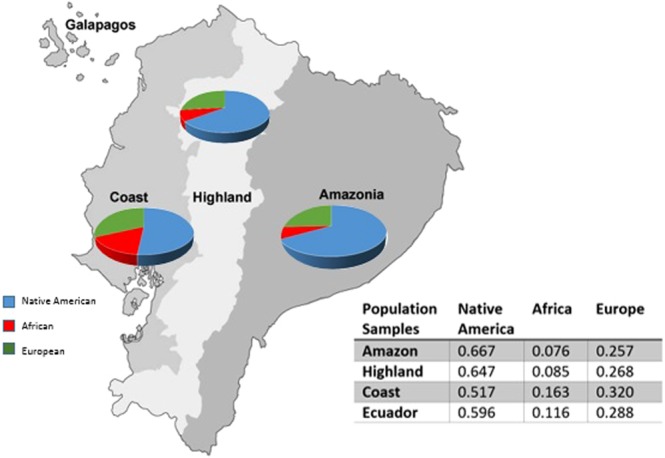
The proportion of Ecuadorian population origin divided into three continental regions. Values were obtained with STRUCTURE v2.3.4 (k = 3, assuming migration model).

The ancestry analysis estimation is presented according to each Ecuadorian region in Fig. 2. By joining the regions as the Ecuadorian population, the composition is as follows: Native American with 59.6%, European with 28.8% and African with 11.6% (Fig. 2).

STRUCTURE data analysis of the Ln P(D) by Evanno method^31^ yielded K = 3 as a result. Supporting the initial inference according to the historical formation of the Ecuadorian population due to the tri-hybrid contribution of Native Americans, Europeans and Africans.

**Figure 3:**
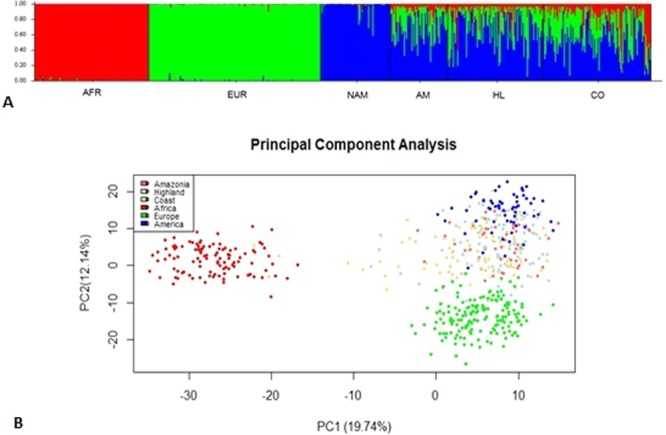
Admixture analysis. (**A**) Bar plot of the composition of the Ecuadorian population compared with the reference population (AFR: Africa; EUR: Europe, NAM: Native America, AM: Amazonia, HL: Highlands, CO: Coast) (k = 3, assuming migration model). (**B**) Principal component analysis of the Ecuadorian and reference populations.

The admixture analysis results are concordant with the genetic distances. Native American ancestry is the main composition of the mestizo Ecuadorian population, with values higher than 51%. Moreover, the African contribution is the least predominant (<16.3% in the Coast) in the analysis for the three regions (Fig. 3A).

Principal component analysis (PCA) allowed us to visualize the variance in the Ecuadorian data regarding the three reference populations (Africans in red, Europeans in green and Native Americans in blue). The two first principal components represent a total of 31.88% of the variance in the dataset and permitted a clear spatial separation of the reference population in three clusters and one cluster in the middle of Europeans and Native Americans (mainly in Native American group) representing the Ecuadorian regions (Amazonia in pink, Highlands in light blue and Coast in yellow) (Fig. 3B).

Furthermore, because we applied a random sampling with a self-identification as a mestizo, we found some individuals with a different prevalent ancestry. Figure 3B shows that three Coast individuals are mainly of African ancestry; for instance, when looking at the composition, one individual has an African proportion of 82.3%, a European proportion of 7.5%, and a Native American proportion of 10.2%. Moreover, there are some individuals from the Coast, and the Highlands are mainly located in the European cluster; as an example, looking at the composition, one individual from the Coast shows 88% European, 5.2% African, and 6.7% Native American.

### Comparative analysis

After the bibliographic revision of previously reported data on the origin of the Ecuadorian population, there were nine scientific articles that used different genetic markers applied to elucidate it.

**Figure 4:**
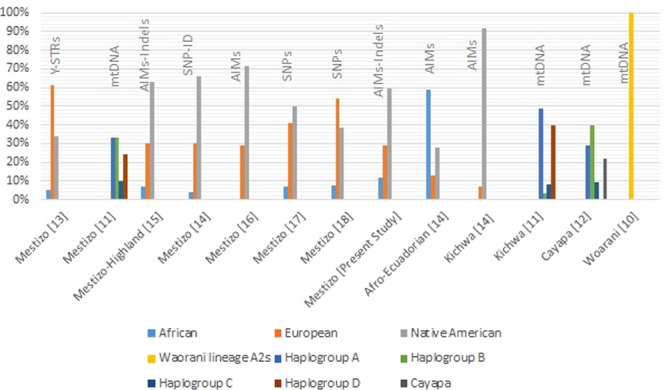
Comparative information of the proportions of each population with the respective method used and the reference population.

The review was based on the public scientific information, and there were a limited amount of published papers about the ancestral characterization of Ecuadorians, but while searching, we saw that there were more papers available regarding genetic markers for forensic identification purposes. Figure 4 compares the ancestral proportion in percentage of each publication found.

When analyzing the ancestral composition percentages, the results are heterogeneous, as shown in Fig. 4. The studies from Zambrano *el al*.^15^, Santangelo *et al*.^14^ and the present study have the closest proportions reported for African (SD = 0.039), Europeans (SD = 0.008) and Native Americans (SD = 0.031).

## Discussion

The main objectives of this study were to infer the ancestry proportions of 240 Ecuadorian samples divided into three continental regions and to compare the results with previously reported data. To achieve this goal, we used a panel of 46 Ancestry Informative InDel markers to corroborate the advantages of their use over other commonly used genetic markers, such as SNPs: (a) the length of the InDel polymorphisms can be genotyped by fragment size separation, while SNP detection requires more complex sequencing methods^22,38,39^; (b) the InDels approach is easy to use and is time and cost effective, reducing the genotyping time compared with AIM-SNPs due to its potential of multiplexing by amplifying it in a single reaction^22,38,39^; (c) the workflow reduces the manipulation of samples, minimizing the number of variables that affect the results, for example, contamination risks or sample mix-ups^22,38,39^; (d) the potential of use in forensic genetics, such as a tool in criminal investigation, because its results could indicate the ancestry of the donor and help direct the case under analysis^22,38,39^.

While there are various studies on genetic markers commonly used in forensic identification analysis in Ecuador^40–45^, the study of ancestral identification markers in the Ecuadorian population is underrepresented in scientific papers.

InDels showed low allele frequency differentials between Ecuadorian regions: δ < 0.178 among Amazonia- Highlands, δ < 0.194 among Amazonia-Coast and δ < 0.14 among Highlands-Coast. Moreover, Hardy-Weinberg analysis did not show any significance, which suggests that there is no process that affects the conditions of Hardy-Weinberg equilibrium^46,47^.

Additionally, the pairwise linkage disequilibrium test detected one significant association within MID-2256 and MID-94 in the Coast population. Hence, while looking at the physical distance between them, it was observed that a significant distance does not exist: MID-2256 is located on chromosome 22, position 41.04 Mb; and MID-94 is also on chromosome 22, but in position 42.55 Mb. This linkage may occur due to the segregation caused by ancestry blocks of the mestizo population that do not allow for adequate recombination^22,37,48^.

The genetic distances F_ST_ between Ecuadorian regions are low, but they are statistically significant. The F_ST_ among Ecuadorian regions and European and African reference populations increase, while the geographic distance from the Coast increases, and the genetic distances with Native American decreases. Moreover, the ancestral characterization percentages are in accordance with the genetic distances for each region: Amazonia (66.7% Native American, 7.6% African and 25.7% European), the Highlands (64.7% Native American, 8.5% African and 26.8% European) and the Coast (51.7% Native American, 16.3% African and 32% European). These results are concordant with the history of Ecuadorian settlement, where the Europeans arrived with African slaves along the coast to later enter the Ecuadorian territory^1,2,5^.

Comparing the ancestral proportions among regions, there are differences that are concordant with the history: Amazonia exhibits greater proportions of Native American origin (66.7%) than the other two regions, which is explained by the number of different indigenous nationalities (Nacionalidad Kichua de la Amazonia, Nacionalidades Siona-Secoya, Nacionalidad A’ICofán, Nacionalidad Waorani, Nacionalidad Andora, Nacionalidad Shiwiar, Nacionalidad Zápara, Nacionalidad Shuar-Achuar and Nacionalidad Quijos) and because until the 1950s, Amazonia was habited principally by indigenous populations^49^. Moreover, the Coast has greater proportions of African ancestry (16.3%) than the other Ecuadorian regions, which was elucidated by a study about an important Afro settlement in Esmeraldas that has its origins based on the arrival of African slaves in 1553 to the coast of that province and due to the group of African slaves that was brought from Colombia in the XVIII century^49^.

It has been reported that there is variation in the proportions of European, African and Native American ancestry among Latin America. Contrasting our results with other available data from America, a study in the Mexican population reported greater Native American ancestry (~75)^50^, which is consistent with the historical records of these populations. Other countries, such as Puerto Rico and the Dominican Republic, reported greater African ancestry (23.6% and 41.8% African, respectively, SDs 12% and 16%, respectively)^18^. In Colombia, which is located on the northern Ecuador border, heterogeneous ancestral proportions were reported depending on the region and the population under analysis, but the results of the present study show low differentiation (F_ST_ = 0.014, p < 5e-05)^36^. In Brazil, the results are also mixed, depending on the population, and when compared with the Ecuadorian population, it showed moderate differentiation (F_FST_ = 0.054, p < 5e-05)^19^ (Fig. S1). The differences could be explained by the geographic distances between the countries.

Additionally, comparing the results of the present study with other available data in the Ecuadorian population, it showed the following:

1. The analysis of the Y chromosome in mestizos showed a greater proportion of European ancestry (61%), followed by Native American ancestry (34%) and African ancestry (5%). The results are because of male inheritance patterns of the Y chromosome^13^;
2. Autosome analysis always shows that the Native American composition is the highest in all the studies: 59.6% (present study), 63.10%^15^, 65.8%^14^, 71.2%^16^, 50.10%^17^ and 38.8%^18^; the different percentages (SD 11.78%) could be due to the sampling procedures because some of the reported studies did not specify the region or the sample where the population under study was from^14–16^. For instance, Santangelo *et al*. (2017) reported that the mestizo population samples were collected at a university located in Quito without specifying the geographic origin of the individuals^14^; another study by Poulsen *et al*. (2011) specified that the samples were collected in Quito, yet no individual origin is provided^16^.
  Thus, the sampling procedure could explain the differences in percentages with the present study because we take into account all the Ecuadorian regions while they specified sampling only in Quito, which is located in the Highland region, but the studies reported an approximation with other previously reported studies of the mestizo highland population^15^. There were two studies using SNPs in 19 and 6 samples in which the percentage of European origin was 40.8%^17^ and 53.90%^18^, which is more than a 10% difference than the other publications (SD 10.78%), 38.8% (present study), 30.3%^15^, 30.1%^14^, 28.8%^16^, which could also be explained by the sample size used of the studies because a study with a small sample size is going to estimate parameters with poor precision or be unable to detect differences between groups^51^. Lastly, African ancestry is similar between the studies (SD 2.7%): 7.30% in the present study, 6.60%^15^, 4%^14^, 6.80%^17^ and 7.30%^18^. Moreover, taking into account only the papers with more than 100 individuals^14–16^, the standard deviation is 4.8%.
3. By using mitochondrial DNA as markers for mestizo and indigenous ancestral characterization, the results correspond to haplogroups A, B, C and D from Native American ancestry, again due to the inheritance patterns^10–12^.

In conclusion, our study demonstrated the use of 46 AIMs-InDels as an alternative method to measure the ancestry proportions in Ecuador and its relevant implications for understanding and corroborating the history and demography of Ecuador. The population admixture in Ecuador started with the arrival of Europeans along with African slaves; however, the Native American ancestry represents the prevalent genetic composition with more than 51%. The autosomal AIM-InDels, mtDNA and Y chromosome information presented support the historical records with a prevalence of Native American but influenced by European (Y chromosome prevalence) and African composition.

## Supplementary information

Figure S1, Table S1

## Data Availability

The datasets generated during the current study are available from the corresponding author on request.
