# Supplementary material for: The three-hybrid genetic composition of an Ecuadorian population using AIMs-InDels compared with autosomes, mitochondrial DNA and Y chromosome data
Source: Sci Rep. 2019 Jun 25;9:9247. doi: 10.1038/s41598-019-45723-w (PMC6592923; doi:10.1038/s41598-019-45723-w)
Supplement: Supplementary file 1 — Figure S1, Table S1 [file 41598_2019_45723_MOESM1_ESM.pdf]

The three-hybrid genetic composition of an Ecuadorian population using AIMs-InDels compared with autosomes, mitochondrial DNA and Y chromosome data.

Ana Karina Zambrano[1*], Aníbal Gaviria[2*], Santiago Cobos[2], Carmen Gruezo[2], Cristina Rodríguez[2], Isaac Armendáriz-Castillo[1], Jennyfer García-Cárdenas[1], Santiago Guerrero[1], Andrés López-Cortés[1], Paola E. Leone[1], Andy Pérez-Villa[1], Patricia Guevara-Ramírez[1], Verónica Yumiceba[1], Gisella Fiallos[2], Margarita Vela[2], César Paz-y-Miño[1]

[1]Centro de Investigación Genética y Genómica, Facultad de Ciencias de la Salud Eugenio Espejo, Universidad UTE, Av. Mariscal Sucre and Mariana de Jesús, Block I, Quito 170129, Ecuador.
[2]Laboratorio de Genética, Centros Médicos Especializados Cruz Roja Ecuatoriana, Papallacta oe1-66, Quito 170512, Ecuador.

[*]Ana Karina Zambrano y Aníbal Gaviria contributed equally to the paper.
Corresponding author:
César Paz y Miño
Address: Centro de Investigación Genética y Genómica, Facultad de Ciencias de la Salud Eugenio Espejo, Universidad UTE, Av. Mariscal Sucre and Mariana de Jesús, Block I, Quito 170129, Ecuador.
Telephone: +593 99 502 6485
E-mail address: cesar.pazymino@ute.edu.ec

Supplementary Figure S1.- Multidimensional scaling plot from F_{ST} between Ecuadorian, Colombian[36] and Brazilian[19 37] populations. Data from Arlequin software and the plot were constructed using STATISTICA v13.

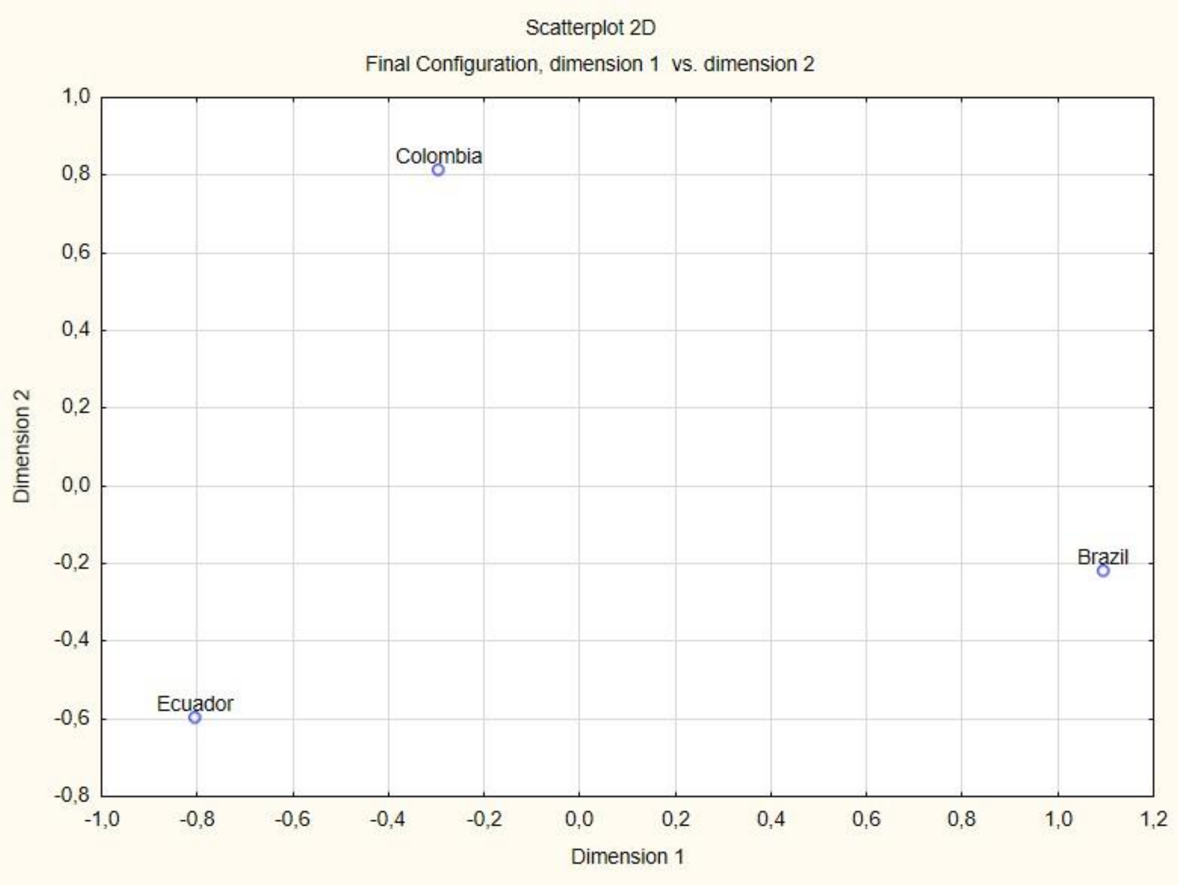

Supplementary Table S1.- Allele frequencies, allele frequency differentials (δ), and observed and expected heterozygosity values for the 46 AIM-InDels in the Ecuadorian population (ECU) joined and divided by region: Amazonia (AM), Highlands (HL), and Coast (CO) and comparison with HGDP-CEPH panel samples from Africa ((AFR), Europe (EUR) and Native America (NAM).

MID	Frequency of allele 1				Frequency of allele 2				Allele frequency differentials (δ)							
	AM	HL	CO	ECU	AM	HL	CO	ECU	ECU/ AFR	ECU/ EUR	ECU/ NAM	AM/HL	AM/CO	HL/CO	Obs. Het (ECU)	Exp. Het (ECU)
MID-1470	0.223	0.119	0.157	0.158	0.777	0.881	0.843	0.842	0.004	0.405	0.127	0.104	0.067	0.037	0.276	0.267
MID-777	0.589	0.523	0.571	0.558	0.411	0.477	0.429	0.442	0.168	0.270	0.286	0.067	0.019	0.048	0.432	0.494
MID-196	0.170	0.165	0.258	0.204	0.830	0.835	0.742	0.796	0.334	0.268	0.141	0.005	0.088	0.093	0.276	0.325
MID-881	0.982	0.915	0.848	0.903	0.018	0.085	0.152	0.097	0.579	0.023	0.089	0.067	0.134	0.066	0.136	0.175
MID-3122	0.973	0.989	0.965	0.975	0.027	0.011	0.035	0.025	0.423	0.016	0.025	0.015	0.009	0.024	0.041	0.048
MID-548	0.152	0.170	0.237	0.193	0.848	0.830	0.763	0.807	0.145	0.003	0.021	0.019	0.086	0.067	0.288	0.313
MID-659	0.268	0.307	0.283	0.288	0.732	0.693	0.717	0.712	0.012	0.184	0.030	0.039	0.015	0.024	0.379	0.411
MID-2011	0.857	0.881	0.798	0.842	0.143	0.119	0.202	0.158	0.680	0.051	0.064	0.024	0.059	0.083	0.226	0.267
MID-2929	0.893	0.920	0.833	0.879	0.107	0.080	0.167	0.121	0.089	0.224	0.098	0.028	0.060	0.087	0.210	0.214
MID-593	0.027	0.034	0.086	0.054	0.973	0.966	0.914	0.947	0.642	0.048	0.046	0.007	0.059	0.052	0.058	0.101
MID-798	0.286	0.290	0.278	0.284	0.714	0.710	0.722	0.716	0.011	0.358	0.167	0.004	0.008	0.012	0.412	0.407
MID-1193	0.054	0.119	0.126	0.107	0.946	0.881	0.874	0.893	0.071	0.009	0.034	0.066	0.073	0.007	0.181	0.191
MID-1871	0.366	0.341	0.298	0.329	0.634	0.659	0.702	0.671	0.248	0.003	0.040	0.025	0.068	0.043	0.403	0.443
MID-17	0.839	0.767	0.717	0.763	0.161	0.233	0.283	0.237	0.032	0.425	0.135	0.072	0.122	0.050	0.325	0.362
MID-2538	0.161	0.170	0.232	0.193	0.839	0.830	0.768	0.807	0.193	0.301	0.130	0.010	0.072	0.062	0.329	0.313
MID-1644	0.473	0.511	0.551	0.519	0.527	0.489	0.449	0.481	0.290	0.434	0.324	0.038	0.077	0.039	0.469	0.500
MID-3854	0.045	0.040	0.177	0.097	0.955	0.960	0.823	0.903	0.651	0.084	0.089	0.005	0.132	0.137	0.160	0.175
MID-2275	0.277	0.364	0.348	0.337	0.723	0.636	0.652	0.663	0.149	0.242	0.186	0.087	0.072	0.015	0.436	0.448
MID-94	0.536	0.580	0.561	0.562	0.464	0.420	0.439	0.438	0.491	0.407	0.086	0.044	0.025	0.019	0.481	0.493
MID-3072	0.982	0.972	0.864	0.930	0.018	0.028	0.136	0.070	0.830	0.010	0.047	0.011	0.119	0.108	0.091	0.130
MID-772	0.759	0.778	0.823	0.792	0.241	0.222	0.177	0.208	0.151	0.176	0.011	0.019	0.064	0.045	0.325	0.330

MID-2313	0.750	0.682	0.556	0.646	0.250	0.318	0.444	0.354	0.536	0.399	0.084	0.068	0.194	0.126	0.428	0.458
MID-397	0.429	0.443	0.495	0.461	0.571	0.557	0.505	0.539	0.310	0.305	0.117	0.015	0.066	0.052	0.551	0.498
MID-1636	0.938	0.915	0.843	0.891	0.063	0.085	0.157	0.109	0.743	0.106	0.101	0.023	0.094	0.071	0.193	0.195
MID-51	0.804	0.801	0.662	0.745	0.196	0.199	0.338	0.255	0.688	0.103	0.122	0.002	0.142	0.140	0.362	0.381
MID-2431	0.277	0.250	0.136	0.210	0.723	0.750	0.864	0.790	0.158	0.099	0.024	0.027	0.140	0.114	0.296	0.332
MID-2264*	0.295	0.216	0.268	0.255	0.679	0.767	0.707	0.722	0.093	0.210	0.122	0.079	0.027	0.052	0.436	0.414
MID-2256	0.518	0.642	0.510	0.560	0.482	0.358	0.490	0.440	0.560	0.338	0.276	0.124	0.008	0.132	0.527	0.494
MID-128	0.196	0.261	0.268	0.249	0.804	0.739	0.732	0.751	0.225	0.232	0.202	0.065	0.071	0.006	0.399	0.375
MID-15	0.563	0.585	0.616	0.593	0.438	0.415	0.384	0.407	0.398	0.140	0.243	0.023	0.054	0.031	0.428	0.484
MID-2241	0.080	0.142	0.253	0.173	0.920	0.858	0.747	0.827	0.641	0.120	0.103	0.062	0.172	0.110	0.247	0.287
MID-419	0.670	0.597	0.641	0.632	0.330	0.403	0.359	0.368	0.287	0.178	0.069	0.073	0.028	0.045	0.457	0.466
MID-943	0.491	0.540	0.571	0.541	0.509	0.460	0.429	0.459	0.365	0.250	0.143	0.049	0.080	0.031	0.465	0.498
MID-159	0.786	0.716	0.717	0.733	0.214	0.284	0.283	0.267	0.019	0.163	0.001	0.070	0.069	0.001	0.420	0.393
MID-2005	0.375	0.375	0.379	0.377	0.625	0.625	0.621	0.623	0.348	0.294	0.052	0.000	0.004	0.004	0.457	0.470
MID-250	0.536	0.494	0.601	0.547	0.464	0.506	0.399	0.453	0.277	0.168	0.136	0.041	0.065	0.107	0.502	0.497
MID-1802	0.054	0.045	0.091	0.062	0.946	0.955	0.909	0.938	0.481	0.059	0.062	0.009	0.037	0.046	0.124	0.117
MID-1607	0.473	0.392	0.379	0.405	0.527	0.608	0.621	0.595	0.019	0.244	0.150	0.081	0.094	0.013	0.432	0.483
MID-1734	0.589	0.625	0.606	0.609	0.411	0.375	0.394	0.391	0.243	0.226	0.234	0.036	0.017	0.019	0.453	0.477
MID-406	0.643	0.688	0.621	0.650	0.357	0.313	0.379	0.350	0.602	0.173	0.053	0.045	0.022	0.066	0.469	0.456
MID-1386	0.545	0.466	0.399	0.457	0.455	0.534	0.601	0.543	0.319	0.239	0.309	0.079	0.146	0.067	0.461	0.497
MID-1726	0.634	0.665	0.636	0.646	0.366	0.335	0.364	0.354	0.470	0.054	0.112	0.031	0.002	0.028	0.461	0.458
MID-3626	0.205	0.216	0.222	0.216	0.795	0.784	0.778	0.784	0.092	0.483	0.177	0.011	0.017	0.006	0.276	0.339
MID-360*	0.723	0.722	0.667	0.700	0.268	0.273	0.323	0.292	0.109	0.164	0.177	0.002	0.057	0.055	0.444	0.426
MID-1603	0.670	0.540	0.561	0.578	0.330	0.460	0.439	0.422	0.497	0.189	0.242	0.130	0.109	0.021	0.498	0.489
MID-2719	0.598	0.420	0.485	0.488	0.402	0.580	0.515	0.512	0.040	0.143	0.019	0.178	0.113	0.064	0.490	0.501

*Markers that present an allele 3: Frequency=1-(frequency allele 1 + frequency allele2)
